# Supplementary material for: Splice-Junction-Based Mapping of Alternative Isoforms in the Human Proteome
Source: Cell Rep. Author manuscript; Available in PMC 2020 Jan 15. (PMC6961840; doi:10.1016/j.celrep.2019.11.026)
Supplement: 2 [file NIHMS1546469-supplement-2.zip › DF1/PXD010154/Colon/Colon_6_PPA1_AIVDALPPPCESACTVPTDVDKWFHHQKM.pdf]

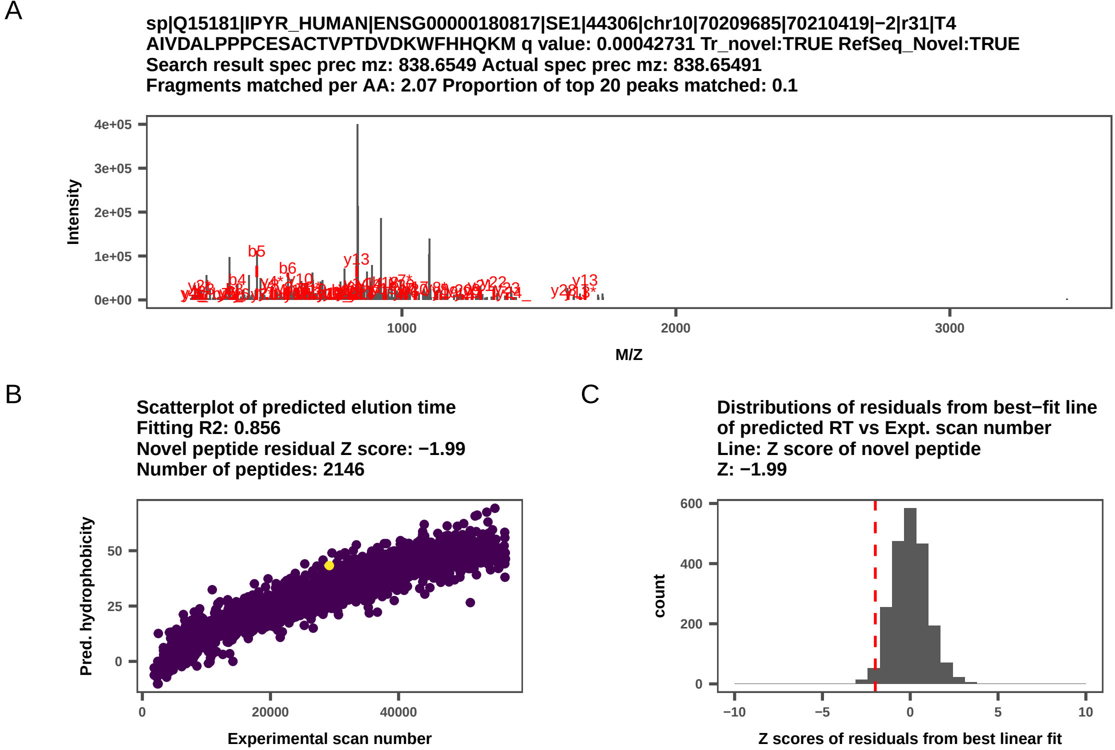
A
sp|Q15181|IPYR_HUMAN|ENSG00000180817|SE1|44306|chr10|70209685|70210419|−2|r31|T4
AIVDALPPPCESACTVPTDVDKWFHHQKM q value: 0.00042731 Tr_novel:TRUE RefSeq_Novel:TRUE
Search result spec prec mz: 838.6549 Actual spec prec mz: 838.65491
Fragments matched per AA: 2.07 Proportion of top 20 peaks matched: 0.1
Intensity
M/Z
B
Scatterplot of predicted elution time
Fitting R2: 0.856
Novel peptide residual Z score: −1.99
Number of peptides: 2146
Pred. hydrophobicity
Experimental scan number
C
Distributions of residuals from best−fit line
of predicted RT vs Expt. scan number
Line: Z score of novel peptide
Z: −1.99
count
Z scores of residuals from best linear fit
